# Children’s Blood Lead Seasonality in Flint, Michigan (USA), and Soil-Sourced Lead Hazard Risks

**DOI:** 10.3390/ijerph13040358

**Published:** 2016-03-25

**Authors:** Mark A.S. Laidlaw, Gabriel M. Filippelli, Richard C. Sadler, Christopher R. Gonzales, Andrew S. Ball, Howard W. Mielke

**Affiliations:** 1 Centre for Environmental Sustainability and Remediation (EnSuRe), School of Science, Royal Melbourne Institute of Technology (RMIT) University, PO Box 71, Bundoora, Victoria 3083, Australia; andy.ball@rmit.edu.au; 2 Department of Earth Sciences and Center for Urban Health, Indiana University—Purdue University Indianapolis (IUPUI), Indianapolis, IN 46202, USA; gfilippe@iupui.edu; 3 Department of Family Medicine, College of Human Medicine, Michigan State University, Flint, MI 48502, USA; sadlerr@msu.edu; 4 Department of Pharmacology, Tulane University School of Medicine, New Orleans, LA 70112, USA; cgonza3@tulane.edu (C.R.G.); hmielke@tulane.edu (H.W.M.)

**Keywords:** Flint, water, lead exposure, soil, seasonality, blood lead, lead poisoning

## Abstract

In Flint; MI; USA; a public health crisis resulted from the switching of the water supply from Lake Huron to a more corrosive source from the Flint River in April 2014; which caused lead to leach from water lines. Between 2010 and 2015; Flint area children’s average blood lead patterns display consistent peaks in the third quarter of the year. The third quarter blood lead peaks displayed a declining trend between 2010 and 2013; then rose abruptly between the third quarters of 2013 from 3.6% blood lead levels ≥5 µg/dL to a peak of about 7% in the third quarter of 2014; an increase of approximately 50%. The percentage of blood lead level ≥5 µg/dL in the first quarter of 2015 then dropped to 2.3%; which was the same percentage as the first quarter of 2014 (prior to the Flint River water source change). The Flint quarterly blood lead level peak then rose to about 6% blood lead levels ≥ 5 µg/dL in the third quarter of 2015; and then declined to about 2.5% in the fourth quarter of 2015. Soil lead data collected by Edible Flint food collaborative reveal generally higher soil lead values in the metropolitan center for Flint; with lower values in the outskirts of the city. The questions that are not being asked is why did children’s blood lead levels display a seasonal blood lead pattern before the introduction of the new water supply in Flint; and what are the implications of these seasonal blood lead patterns? Based upon previous findings in Detroit and other North American cities we infer that resuspension to the air of lead in the form of dust from lead contaminated soils in Flint appears to be a persistent contribution to lead exposure of Flint children even before the change in the water supply from Lake Huron to the Flint River.

## 1. Introduction

Lead is a pernicious and widespread pollutant, with a very clear and incontrovertible link between exposure and human disease. The environmental sources of exposure in the modern landscape are many, including largely legacy sources such as leaded gasoline [1], lead-based paints [2], lead leached from water pipes and solder [3], and industrially-sourced lead [4]. Most of these legacy sources were removed from the consumer stream after regulations in the latter part of the 20th century, although the damage was already done, in a sense, as the insolubility of lead in natural systems resulted in the accumulation and concentration of lead in surface soils and dust over decades. Older inner city areas are particular hotspots for legacy lead and commonly exhibit the highest levels of lead exposure in urban populations.

The primary pathway for the human exposure and uptake of lead in children is via inadvertent ingestion of lead-contaminated soils and dust, and subsequent absorption in the intestines [5]. Exposure and uptake are age dependent; toddlers and small children ingest much more dirt than adults [6], and their underdeveloped intestines also absorb as much as 50% of the lead they inadvertently ingest [7]. Elevated lead in children leads to lowered Intelligence Quotient (IQ), learning disorders, and behavioral problems [8,9]. These impacts are also largely age dependent, as neural development is most rapid in young children, and the presence of lead in blood during this time interferes with proper neuron formation [10]. Furthermore, much of the lead initially present in blood is stored for years in bone, which becomes a long-term chronic source of lead back into the blood stream even after a child might be removed from a lead-rich environment; indeed, there is even some indication of decreased cognitive function in elderly osteoporotic patients from the increased release of lead into the bloodstream due to rapid bone loss [11]. A review of the impacts of low level lead concentrations in children has been compiled by the United States Department of Health and Human Services National Toxicology Program [12], United States Environmental Protection Agency [13], and the United States Agency for Toxic Substances and Disease Registry (ATDSR) [14].

This paper is a review of our current understanding of the nature of lead exposure and uptake in children, with a particular focus on the role of dust as a dominant source of exposure to urban children. Additionally, in light of the Flint Michigan water crisis starting in 2015, and using related evidence of widespread lead exposure from drinking water supplies, we assess temporal patterns in lead exposure in Flint compared with national and Michigan data. We find that lead exposure from soil and dust sources in Flint before the water crisis was already high and predictable from seasonal patterns. The Flint water lead crisis should be viewed from a larger perspective; although the water problem can be addressed through targeted efforts to improve the condition of the water network, the soil-sourced lead has and will continue to expose millions of children throughout the United States unless we take action to creatively address this problem.

## 2. Background

### 2.1. Lead Contamination of Urban Soils

Inner city soils in most major municipalities of the United States (and globally) are contaminated with lead, primarily from past use of lead in gasoline and the deterioration of exterior lead based paints, as well as from lead smelters and industry [15]. The amount of lead contamination in the soil of inner city areas is proportional to historical traffic flow volumes during the period when lead was used in gasoline [16]. This reservoir of highly bioaccessible lead in urban soil and dust derived from that soil is concentrated in the top 20–30 cm of the surface soil [17] where it is available to be resuspended to the air in the dry summer periods. The exposure pathway of lead in soil and dust consists of track-in via soil material attached to shoes [18,19,20], pets tracking lead indoors on their fur and paws [21], soil resuspension [22,23,24,25], and direct contact with soils during the summer time when the weather is favorable and children spend more time outdoors.

### 2.2. Association Between Soil Lead and Blood Lead

In New Orleans, Louisiana, Zahran *et al.* [26] combined two extensive data sets: (i) 5467 surface soil lead samples collected from 286 census tracts; (ii) geo-referenced blood lead data for 55,551 children in metropolitan New Orleans, USA. The results indicated that children’s blood lead levels are spatially associated with soil lead levels. Seventy-seven percent of the variation in children’s blood lead was explained by the four independent soil lead sample location variables—house-side, residential street-side, busy street-side, and open-space. Importantly, this study adds to a very small number of large sample studies that have examined the spatial relationship between soil lead and blood lead [27,28,29]. The dose response relationship between soil lead and blood lead using the United States Environmental Protection Agency’s (USEPA) Integrated Exposure Uptake Biokinetic (IEUBK) model is shown in Davis *et al.* [30] and Laidlaw and Taylor [31].

### 2.3. Atmospheric Lead and Soil Seasonality

Contemporary atmospheric concentrations of lead reach maxima during summer and autumn in many United States cities, including Pittsburgh [23] Birmingham [23], Detroit [23,24], Washington, DC [32,33], Boston [34], Milwaukee [35], New York [36], New Jersey [37], and Chicago [38]. Atmospheric soil has also been shown to peak in the dry months and is highly correlated with atmospheric lead concentrations. This has been observed in Detroit, Pittsburgh, Birmingham Chicago [23], and throughout the United States [39].

### 2.4. Blood Lead Seasonality Studies

**Figure 1:**
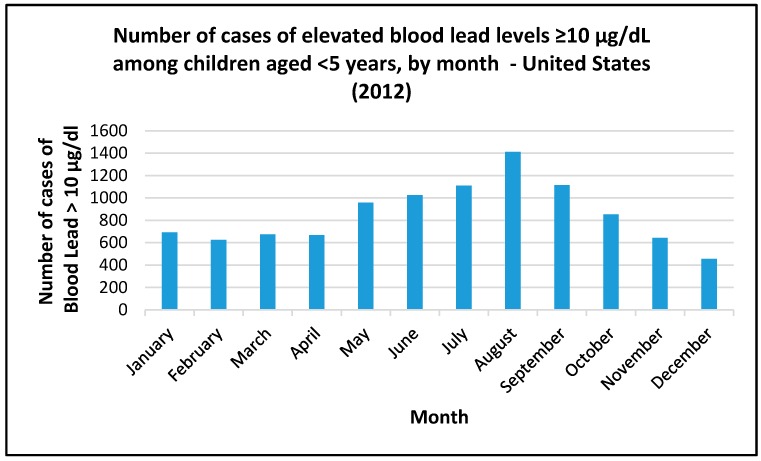
Number of cases of elevated blood lead levels ≥10 micrograms per decilitre among children aged <5 years, by month in 2012 in the United States (source of data CDC, 2016 [40]).

Blood lead levels in the United States display a predictable pattern with peaks in late summer/early autumn during dry periods of the year [40] (Figure 1).

This same cyclical pattern has been observed in Detroit [24], Lansing [41,42], Indianapolis [22], Milwaukee [35,43], Boston [34], Los Angeles [44], Syracuse [45], New Jersey [37], and the State of New York [46].

Seasonal patterns in children’s blood lead levels in Indianapolis, Syracuse and New Orleans were predicted using variables such as soil moisture, particulate matter ≤10 µm (PM_10_), wind speed, and temperature [22]. These variables explained between 59 and 87 percent of the variation in blood lead levels. The conceptual model at the time was that when temperature is high and evapotranspiration maximized, soil moisture decreases, and soil-derived dust is deposited. Under these combined weather conditions, lead-enriched PM_10_ dust disperses in the urban environment and causes elevated lead dust loading causing elevated blood lead levels in the dry summer and autumn months. The limitation of this study was that the authors did not have air lead or soil lead values to correlate with the children’s blood lead levels.

The association between soil lead resuspension and exposure to children has been revealed in a number of studies [39]. In 2012, Laidlaw *et al.* [23] observed that atmospheric lead and soil were highly correlated with peaks in summer and autumn in the cities of Birmingham, Chicago, Detroit, and Pittsburgh. Later in 2013, Zahran *et al.* [24] obtained a dataset of air lead and soil measurements from the United States Environmental Protection Agency’s (EPAs) Interagency Monitoring of Protected Visual Environments (IMPROVE) database [47] in Detroit and also the blood lead dataset of Detroit’s children. Atmospheric lead levels and children’s blood lead levels were highly correlated with peaks reoccurring between 2000 and 2009 in a cyclical pattern during the hot and dry months of the summer and autumn. Further research in Australia in 2014 showed the same summer atmospheric lead peak and indicated that where soil lead levels were highest the lead loading depositional rates were highest, especially during the summer period [25]. Furthermore, a re-evaluation of a large study in Sydney, NSW (Australia), concluded that higher lead contents in summer in the interior of homes in Sydney may arise from the action of residents opening and closing doors and windows in the summer and the higher temperatures giving rise to dust that enters the residence [48].

### 2.5. Lead in Drinking Water as a Source of Blood Lead Seasonality

Drinking water obtained from lead water service lines has been shown to display seasonal variations with concentrations that rise during the summer when the temperature rises. Shock (1990) [49] suggested that studies by others [50,51,52] indicated that the net effect of temperature for the waters studied was an approximately two- to three-fold increase in lead solubility as temperature increased from 5 to 25 degrees Celsius (°C). Furthermore, the amount of increase in lead solubility depended upon the pH of the water and the carbonate content due to the temperature effect on the various dissociation, solubility and complexation reactions [49]. Cartier *et al.* (2011) [53] observed lead levels in water lines at different temperatures in a distribution system with lead service lines. They concluded that a 1 °C rise in water temperature translated roughly to a 5% increase in lead concentrations in flushed samples when temperatures varied between 10 and 23 °C. In Montreal, Canada, Ngueta *et al.* [54] observed that the magnitude of winter-to summer changes in average concentrations of lead corresponded to 6.55 μg/L in homes served by lead service lines and only 0.30 μg/L in homes without lead service lines. Furthermore, they observed that for stagnant samples, the average concentrations of lead reached 10.55 μg/L in homes with lead service lines. Using the IEUBK model they calculated that the maximum contribution to blood lead during the summer could result in a rise of approximately 1 µg/dL. They also calculated that the probability of BLLs ≥5μg/dL due to winter-to-summer changes in water lead levels was increased from <5% (in winter) to about 20% (in summer) in children aged 0.5–2 years. Thus, these results suggest that lead originating from drinking water from areas with leaded water service lines can contribute to the seasonal rise in blood lead levels in a city.

### 2.6. Other Potential Sources of Blood Lead Seasonality

Other potential sources of blood lead seasonality include the lead dust created from the opening and closing of windows which contain lead paint. This has the potential to release lead from the deteriorated lead paint due to the friction of opening and closing windows. Repainting and renovation activities are more common in summer and also have the potential to release lead dust particles [55].

### 2.7. Blood Lead Levels in Michigan and the United States

Jain (2016) [56] assessed the distribution of blood lead levels in children across the United States using data collected by the National Health and Nutrition Examination (NHANES) for the time period of 2003–2010. For children aged 1–5 years old, approximately 7.8% of Non-Hispanic Blacks, 1.8% of Mexican Americans and 2.4% of Non-Hispanic whites exhibited blood lead levels exceeding 5 µg/dL.

The Michigan Department of Environmental Quality reported in 2013 that in the city of Detroit, Genesee County (containing the city of Flint) and the State of Michigan, 8%, 2.2%, and 3.9% of children tested exhibited blood lead levels exceeding 5 µg/dL, respectively [57].

It must be noted that in some neighborhoods and regions of inner cities, a much larger percentage of children can have blood lead levels exceeding 5 µg/dL. For example, in some neighborhoods of Indianapolis, 27% of children exhibit blood lead levels exceeding 5 µg/dL [58].

**Figure 2:**
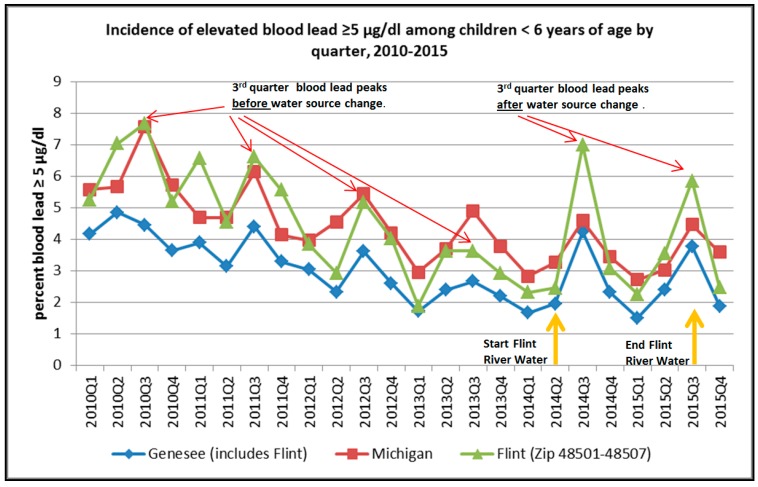
Incidence of blood lead ≥5 µg/dL among children <6 years of age by quarter from 2010 to 2015 (source of data—MDEQ, 2016 [59]).

In Flint, Michigan (USA), a public health crisis resulted from the switching of the water supply from treated Lake Huron water to untreated corrosive water sourced from the Flint River in April 2014, which caused lead to leach from water lines exposing a portion of the children in the city. The quarterly blood lead levels of children <6 years old in Flint (as well as in the State of Michigan and Genesee County) were compiled between 2010 and 2015 and the percentage of children with blood lead levels ≥5 µg/dL were calculated by the Michigan Department of Environmental Quality (MDEQ). The percentage of children with blood lead levels ≥5 µg/dL in the third quarter of 2013 (before the water source switch) rose from about 3.6% to about 7% in July–September) 2014 (see Figure 2; [59]). This figure clearly shows the seasonal nature of children’s blood lead pattern between 2010 and 2015 with a peak in the third quarter and a trough in the first or second quarter. Research undertaken by local hospital and public health doctors—which sparked international attention on the current situation in Flint—confirmed this change, showing that the rate of elevated blood lead levels among children within the City of Flint significantly increased from 2.4% to 4.9% between 2013 and 2015 [60].

Based on the media reports, an important contributor to the Flint, Michigan, water crisis was the uncertainty by the MDEQ regulators and government officials regarding whether the population blood lead levels were actually showing an increase that deviated from the cyclical seasonal pattern observed during the third quarter (July, August, September) in the years prior to the water change. The questions not being asked by the media are why did children’s blood lead levels display a seasonal pattern before the introduction of the new water supply, and what are the implications of these seasonal blood lead patterns?

## 3. Methods and Results

### 3.1. Quarterly Blood Lead Incidence Trend—Flint, Michigan

The quarterly blood lead incidence data for children < 6 years of age in Michigan, Flint’s urban area (ZIP codes 48501–48507—which include some suburban and rural areas immediately adjacent to the City of Flint) and Genesee County (includes Flint) were extracted from a MDEQ chart [59] using data extraction software WebPlotDigitizer (company, city, country) [61] and are presented in Figure 2. The precision was assessed by measuring the standard deviation from three digitizing events. The standard deviation of the digitizing process ranged between 0.05 and 0.06 percent blood lead levels greater than 5 µg/dL. This blood lead data was generated from blood lead measurements collected from 889,397, 41,819, and 19,557 children aged ≤6 years old in the State of Michigan, Genesee County, and the Flint area between 2010 and 2015, respectively [59]. Additionally, 39,693, 1264, and 872 children aged ≤6 years old had blood lead measurements ≥5µg/dL in the State of Michigan, Genesee County, and the Flint area between 2010 and 2015, respectively [59].

Examination of the data indicates that blood lead generally peaked in the three areas during the third quarters (July–September) of each year. The State of Michigan data indicate that the slope of declining blood levels is steepest between 2010 and 2012 and the slope plateaus between 2013 and 2015. The third quarter blood lead peak in Genesee County displayed a declining trend between 2010 and 2013 and rose from about 2.7% in 2013 to 4.2% blood lead levels ≥5 µg/dL in 2014 and then declines to about 3.8% in 2014.

Correlation of the associations between blood lead levels in the three regions was performed using Spearman Rank Order Correlation tests. Results indicate that the three blood lead curves are strongly correlated.

Table 1 shows that the percentage of children’s blood lead ≥5 µg/dL in Flint is closely associated with the blood lead of the children of the State of Michigan. The correlation of percentage blood lead ≥5 µg/dL of the children of Genesee County overall is even more strongly associated than appearing for the children of Flint. The blood lead data of Flint is nested within the Genesee County blood lead data.

**Figure 3:**
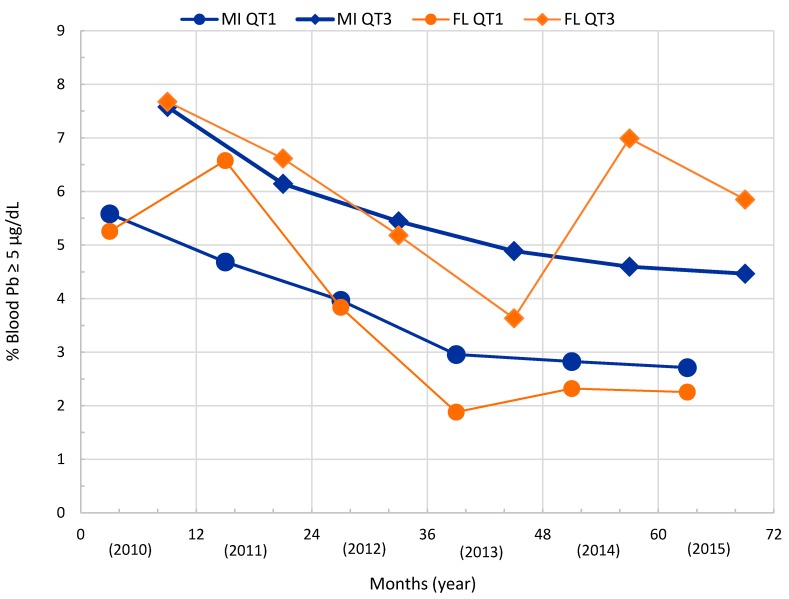
Seasonal differences between Quarter 1 and Quarter 3 for six years of percent blood lead ≥5 µg/dL for the children of the State of Michigan and Flint area children. The data in this figure is the same as shown in Figure 2, but it emphasizes the seasonal differences in the blood lead.

Figure 3 illustrates the seasonality of children’s blood lead levels by comparing the third quarter and first quarter percent ≥5 µg/dL children’s blood lead for Flint compared with the State of Michigan. There is a larger percentage of children’s blood lead ≥5 µg/dL in the third quarter (summer) compared with the first quarter (winter). The figure also displays a declining trend of blood lead for the State of Michigan between 2010 and 2015. In contrast, Flint shows a decline up to the third quarter and a rise in blood lead between the third quarters of 2013 from 3.6% blood lead levels ≥5 µg/dL to a peak of about 7% in the third quarter of 2014, an increase of approximately 50%. The percentage of blood lead level ≥5 µg/dL in the first quarter of 2015 dropped to 2.3%, which was the same percentage as the first quarter of 2014 (prior to the Flint River water source change). The Flint blood lead level rose to about 6% ≥5 µg/dL in the third quarter of 2015.

Statistical analysis of the first quarter and third quarter percent blood lead ≥5 µg/dL results was done by Multi-Response Permutation Procedures for blocked data. This non-parametric test is analogous to a *t*-test for matched pairs [62]. There is a non-significant difference between the State of Michigan and Flint for the first and third quarters percent blood lead ≥5 µg/dL (*p*-values = 0.302 and 0.581, respectively). In the case of Genesee County, compared with the State of Michigan, there is a significant difference between the first quarter and between third quarter percent blood lead ≥5 µg/dL (*p*-values = 0.008 and 0.012, respectively). Significant differences were found when comparing Genesee County to the selected areas of Flint between first quarter and between third quarter percent blood lead ≥5 µg/dL (*p*-values = 0.011 and 0.009, respectively). The State of Michigan’s quarterly percentages of children’s blood lead ≥ 5 µg/dL are similar to the ones seen in Flint. There are smaller percentages of children in Genesee County with blood lead ≥5 µg/dL than in either the State of Michigan or the selected Flint areas.

### 3.2. Soil Lead Data—Flint, Michigan

**Figure 4:**
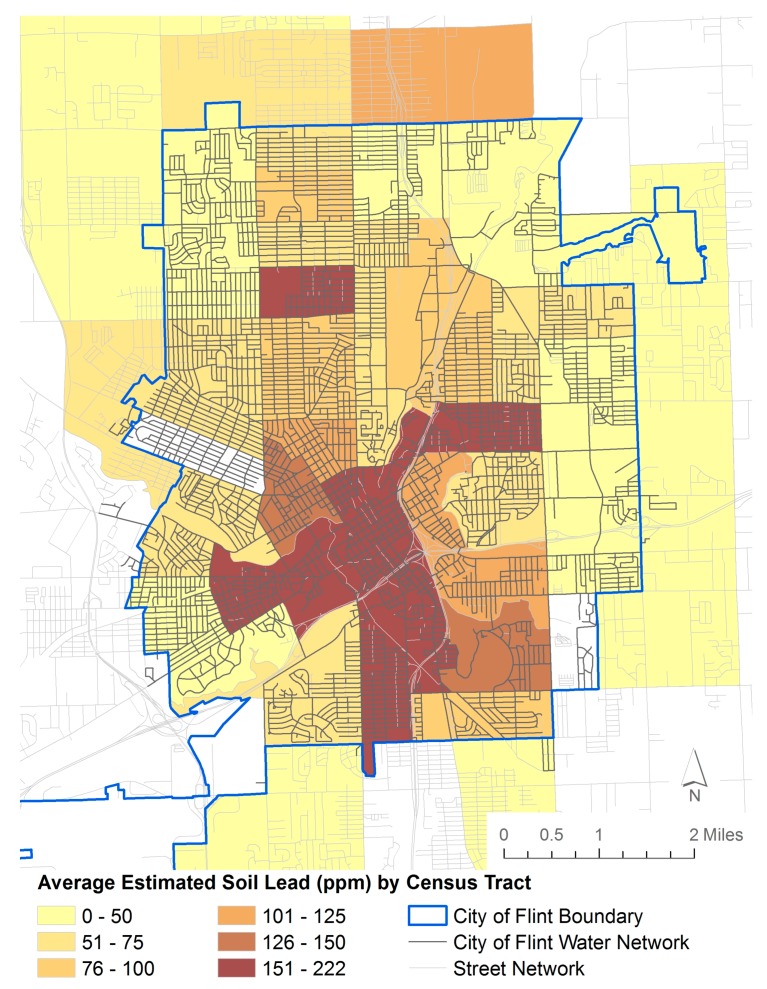
Average estimated soil lead concentration by census tract in Flint, Michigan. The soil lead was derived from extractable lead concentrations via a linear regression model.

This paper integrates bulk composite soil samples previously collected by the Edible Flint local food collaborative [63] throughout the city from 2011 to 2015. Each sample was collected at the start of the spring growing season (typically in the second quarter) from 10 to 15 randomly selected sites at personal residences and community gardens, exclusively in residential neighborhoods. Edible Flint staff and volunteers conducting the sampling were trained to use the Michigan State University soil test protocol, which included: sampling from untilled soils at a depth of 3–4 inches for lawns and 7 inches for gardens, using a metal or plastic hand spade to collect samples, and storing samples in Ziploc or plastic bags. A total of 248 samples were collected. Samples were analyzed by the University of Massachusetts Soil & Plant Tissue Testing Laboratory with Spectroblue ICP analyzers using the Modified Morgan extraction procedure to estimate plant available soil lead [64]. The data was then aggregated by census tract (Figure 4), yielding a typical sample density of 4/census tract.

Because this data set was for agricultural purposes and thus the target was plant-extractable lead in the root zone, we developed a linear calibration model to expected surface soil lead concentrations. This linear model was derived from measured values on a subset of samples. The r^2^ for the calibration was 0.98. Note that these comparisons have been tested in a number of studies, with linear calibration r^2^ ranging from 0.30 [65] to 0.825 [66], indicating relatively high variability. 

These results reveal generally higher soil lead values in the metropolitan center for Flint, with lower values in the outskirts of the city (Figure 4). This result is comparable to the patterns observed in many other urban areas (e.g., 17 and references therein), and is the result of a higher concentration of lead sources in the higher density (both housing and traffic) cores of older cities with significant historic inputs from gasoline, paint, and industry. A comparison of soil lead values and the distribution network for City of Flint water reveals a strong overlap between neighborhoods with high soil lead and city water systems, indicating that both potential sources of exposure need to be taken into account when considering public health risks for lead in this context.

## 4. Discussion

### Blood Lead Seasonality and Flint

Based on our interpretation of blood lead seasonality in urban areas such as Detroit [24] (located approximately 100 km southeast of Flint), we infer that before the change in water supply the blood lead peaks in the third quarters in Flint were being driven by seasonal resuspension of lead dust from contaminated soils into the atmosphere during dry periods. This lead source may have been compounded by contributions from lead leaching from water pipes during the summer. This longer-term view of lead uptake via blood lead levels indicates that the blood lead peaks in the third quarters of 2014 and 2015 may have been driven by a combination of lead from resuspended soil-derived dust and lead newly released from water lines. A paucity of environmental data exists for lead in Flint, and indeed, no air lead values were found in public databases or the literature. The distribution of soil lead from Flint (Figure 4) does reveal elevated values in the metropolitan core and lower values in the outskirts of the city. Indeed, the inner city values were four times higher than those in the outskirts, indicating that elevated soil lead does exist in Flint and that this can be a reservoir for high air lead loading during seasonal resuspension events.

It is important to consider this as not solely a water problem with a clear solution and resulting in a short-term though obviously tragic impact on residents, but a problem that appears to be a water lead, soil-dust lead, and air lead problem. We suggest that the water lead contribution in the third quarter of 2014 and 2015 in Flint is being superimposed on the contribution from soil resuspension. When the contribution of lead from water in Flint is minimized, we suggest that the third quarter blood lead peaks might continue to occur until exposure of children from surficial soil lead is ameliorated.

## 5. Conclusions and Recommendations

Soil lead and its seasonal resuspension and deposition into homes is a major contributor to chronic lead exposure in the United States. Clearly, lead from deteriorating lead-based paint and lead leaching from water pipes are very important sources of exposure, with lead paint especially attributable to acute exposure. However, the constant background of soil-dust lead exposing millions to chronic and persistent lead cannot be “remediated away” by repainting houses or changing water supply chemistry. A failure to isolate soils in urban inner city areas will result in the continued exposure of children during the summer time due to direct contact with lead contaminated soil dust as well as resuspension of lead contaminated soil dust. Selective approaches to the isolation of lead-contaminated surface soils in high-risk urban areas are called for to rid populations of the lead nuisance once and for all [67,68,69].

Ultimately, the very fact that we are identifying these problems *after* the children have already been exposed and potentially *permanent harm has already come* to the health and future reveals a significant failing in the environmental health system, where environmental protection and management is disconnected from public health surveillance systems [60], often with corporate and/or municipal interests lying between the two. Indeed, public policy and systems need to change in order to more adequately integrate and inform issues of urban environmental exposure [70].

One key limitation in our ability to constrain the various sources of lead to children and thus apportion the observed third quarter peak to air and water is that limited data exists for soil lead, atmospheric soil, and atmospheric lead data available in Flint, Michigan. We recommend that in Flint, it is paramount to more comprehensively map the soil lead (and other soil toxins) and measure the seasonal variations in water lead levels, atmospheric lead loading (interior and exterior), and concentration. This will permit a better understanding of the blood lead seasonality phenomenon and will inform measures to reduce the factors driving blood lead seasonality patterns.

## Figures and Tables

**Table 1:** Spearman Rank Order Correlation matrix of percent blood lead ≥5 µg/dL in children of the State Michigan, Genesee County (including Flint) and Flint’s urban area (ZIP 48501–48507).

Location	Genesee	Flint
Michigan	0.877 *	0.816 *
Genesee	-	0.977 *

* *p* < 0.001.
